# The prognostic value of tumor-stroma ratio and a newly developed computer-aided quantitative analysis of routine H&E slides in high-grade serous ovarian cancer

**DOI:** 10.21203/rs.3.rs-3511087/v1

**Published:** 2023-11-14

**Authors:** lilian van wagensveld, Cedric Walker, Kerstin Hahn, Joyce Sanders, Roy Kruitwagen, Maaike van der Aa, Gabe Sonke, Sven Rottenberg, Koen Van de Vijver, Andrew Janowczyk, Hugo Horlings

**Affiliations:** Netherlands Comprehensive Cancer Organization; University of Bern; Roche; The Netherlands Cancer Institute; Maastricht University Medical Centre; Netherlands Comprehensive Cancer Organization (IKNL); NKI; The Netherlands Cancer Institute; Ghent University Hospital; Emory University and Georgia Institute of Technology; The Netherlands Cancer Institute

## Abstract

**Introduction:**

Tumor-stroma ratio (TSR) is prognostic in multiple cancers, while its role in high-grade serous ovarian cancer (HGSOC) remains unclear. Despite the prognostic insight gained from genetic profiles and tumor-infiltrating lymphocytes (TILs), the prognostic use of histology slides remains limited, while it enables the identification of tumor characteristics via computational pathology reducing scoring time and costs. To address this, this study aimed to assess TSR’s prognostic role in HGSOC and its association with TILs. We additionally developed an algorithm, Ovarian-TSR (OTSR), using deep learning for TSR scoring, comparing it to manual scoring.

**Methods:**

340 patients with advanced-stage who underwent primary debulking surgery (PDS) or neo-adjuvant chemotherapy (NACT) with interval debulking (IDS). TSR was assessed in both the most invasive (MI) and whole tumor (WT) regions through manual scoring by pathologists and quantification using OTSR. Patients were categorized as stroma-rich (≥ 50% stroma) or stroma-poor (< 50%). TILs were evaluated via immunohistochemical staining.

**Results:**

In PDS, stroma-rich tumors were significantly associated with a more frequent papillary growth pattern (60% vs 34%), while In NACT stroma-rich tumors had a lower Tumor Regression Grading (TRG 4&5, 21% vs 57%) and increased pleural metastasis (25% vs 16%). Stroma-rich patients had significantly shorter overall and progression-free survival compared to stroma-poor (31 versus 45 months; P < 0.0001, and 15 versus 17 months; P = 0.0008, respectively). Combining stromal percentage and TILs led to three distinct survival groups with good (stroma-poor, high TIL), medium (stroma-rich, high TIL, or; stroma-poor, Low TIL), and poor(stroma-rich, low TIL) survival. These survival groups remained significant in CD8 and CD103 in multivariable analysis (Hazard ratio (HR) = 1.42, 95% Confidence-interval (CI) = 1.02–1.99; HR = 1.49, 95% CI = 1.01–2.18, and HR = 1.48, 95% CI = 1.05–2.08; HR = 2.24, 95% CI = 1.55–3.23, respectively). OTSR was able to recapitulate these results and demonstrated high concordance with expert pathologists (correlation = 0.83).

**Conclusions:**

TSR is an independent prognostic factor for survival assessment in HGSOC. Stroma-rich tumors have a worse prognosis and, in the case of NACT, a higher likelihood of pleural metastasis. OTSR provides a cost and time-efficient way of determining TSR with high reproducibility and reduced inter-observer variability.

## Introduction

Ovarian cancer is the most lethal gynecological malignancy in developed countries.(1) In most cases, the disease is diagnosed at an advanced stage and relapse is common, resulting in 8-year survival of less than 20%.(2) It remains a challenge to identify patients in whom treatment is likely to fail, and reliable biomarkers for predicting treatment failure and survival are urgently needed. In an attempt to address this need, the influence of the tumor microenvironment (TME) and molecular profiles on survival has become a topic of interest, particularly in high-grade serous ovarian carcinoma (HGSOC). (3–5)

While studies on the TME and molecular profiling have changed the way we think about ovarian cancer heterogeneity, (3–5) the translation of the knowledge of the found tumor subtypes into the clinical setting is often limited by the cost and scale of molecular profiling, even more so in developing countries. Besides cost-associated issues, not all samples meet the needed quality or quantity required for such tests. Thus, cost-effective and generally applicable alternatives could accelerate the translation of new research discoveries and the development of quantitative biomarkers for ovarian cancer.

It has often been described that the development and progression of ovarian cancer are influenced by the interaction between tumor cells and the microenvironment, mainly conferred by the tumor stroma.(6) The stroma is composed of the extracellular matrix and connective-tissue cells such as fibroblasts and mesenchymal stromal cells.(7) A higher stromal percentage as reflected in the Tumor-Stroma Ratio (TSR), has been associated with tumor growth, metastasis, chemoresistance, and recurrence in multiple epithelial cancers. Tumor stroma is hypothesized to confer its tumor-promoting effect via different mechanisms such as extracellular matrix remodeling, suppression of immune cells, and alteration of stromal regulatory pathways. (8–13)

Profiling of ovarian cancer based on TSR with the use of Hematoxylin and eosin (H&E) stained slides has been reported infrequently,(14–16) despite its cost-efficiency, DNA independence, and depicted prognostic role in other cancer types. Additionally, studies that have investigated the influence of manually determined TSR have focused on the most invasive (MI) tumor region,(14, 15) while the few studies based on automated image analysis have focused on the whole tumor (WT), limiting comparability between both scoring techniques.(16, 17) Advances in the infrastructure of digital pathology, improvement of deep learning models, and the existence of national databases result in the opportunity to further develop automated image analysis methods. The ability to objectively identify stromal components via computational pathology could enable computer-aided diagnosis(18), complement molecular analysis in a cost-efficient manner, and uncover new therapeutic targets. It also reduces inter-observer variability allowing a more robust categorization of patients. Therefore, in the present study, we aimed to determine the clinical implication of TSR in HGSOC and to develop an accurate image analysis classifier (Ovarian-TSR; OTSR).

## Materials and Methods

### Patient selection.

360 patients with advanced stage (FIGO stage IIb-IV) HGSOC treated with a combination of cytoreductive surgery and chemotherapy from the Netherlands Cancer Institute - Antoni van Leeuwenhoek Hospital (NKI-AVL), Maastricht University Medical Centre (MUMC) and Amsterdam University Medical Centre (AUMC), between January 2008 and December 2015, were included. Of these, 141 patients received primary debulking (PDS) and adjuvant chemotherapy (NKI-AVL n = 52, MUMC n = 30, AUMC n = 59), and the remaining 219 patients neoadjuvant chemotherapy (NACT) followed by interval debulking surgery (IDS) (all NKI-AVL) (Supplementary figure S1A).(19)

Clinical data was extracted from the Netherlands Cancer Registry (NCR), a nationwide registry covering all primary malignancies in the Netherlands, and histopathological data from the Dutch Pathology Registry (PALGA). For the present study, approval of the institutional review boards of the NCR (reference number; K22.367), PALGA (reference number; 2016–82), and NKI-AVL (reference number; CFMPB297), was obtained. Ethical approval was waived according to Dutch legislation. The following parameters were extracted from the NCR; performance status, germline BRCA status, treatment sequence (NACT, NACT-IDS, or PDS), surgery outcome (complete with no visible disease; optimal with ≤ 1 cm residue or sub-optimal with > 1 cm residue), distant metastasis (grouped based on localization as pleural malignant effusion, parenchymal, extra-abdominal lymph node, and “other” if there were metastases in multiple categories), and progression status. Progression of disease was defined in the case of symptoms combined with increased serum CA-125 levels, radiological signs of progression, or histological or cytological confirmation of recurrent disease. Vital status and date of death were obtained via linkage with the municipal population registration.

### Hematoxylin and eosin (H&E) image processing

Representative samples of primary ovarian cancer from formalin-fixed, paraffin-embedded (FFPE) tissue blocks of the 360 patients were obtained from PALGA (Supplementary figure S1B) and were either pretreated or chemo-naïve depending on therapy sequence. All cases underwent central pathological review by three dedicated pathologists (KVdV, HH, JS). Tumor Regression Grade, similar to the Mandard score, of pretreated slides was determined.(20) 4 μm thick tissue sections were taken to perform H&E staining. H&E slides were scanned using an Aperio scanner (Aperio, San Diego, USA) at 40 x yielding digital images with a resolution of 0.25 μm/pixel. All acquired images were anonymized for research purposes.

### Manual TSR scores

Each of the H&E-stained whole slide images (WSI) was evaluated by two pathologists (KVdV, JS), who specialized in gynecologic oncology, while being blinded for patient characteristics and outcome. Two TSR scores were determined; the TSR of the MI area of the tumor and the TSR of the WT area. Slides were scored through the online scoring platform Slidescore (https://www.slidescore.com). Both pathologists selected the MI of each slide with the use of a 4x objective. With a 10x objective, the pathologists chose a part of the sample containing both cancer and stromal cells. Cancer cells had to be present at all borders of the image field. The percentage of cancer and stromal cells were scored while visually excluding necrosis, adipose tissue, psammoma bodies, and vessels. The WT area was determined and scored similarly. The WT area of each slide was selected with the use of a 4x objective and the percentage of cancer cells and stromal infiltrate was scored. The stromal density was evaluated per tenfold percentage (10%, 20%, 30%, etc.). In case of a lack of concordance between both pathologists’ scores, a third pathologist (HMH) was consulted to reach a consensus. Patients were further stratified in stroma-rich (stroma ≥ 50%) or a stroma-poor (stroma < 50%), consistent with previous literature.(15)

### Computational scoring

**Figure 1:**
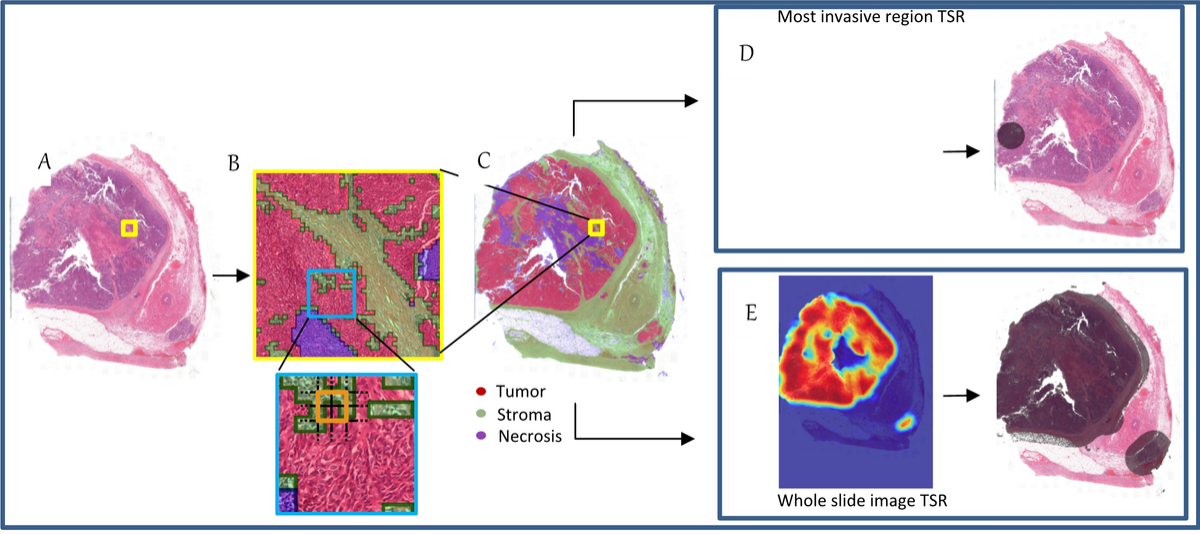
Depiction of the computational scoring method. Determination of the stromal percentage in the most invasive region and whole tumor area based on a whole slide image. Panels A-E, from left to right. A. The whole slide image (WSI). B. Zoomed in WSI showing the segmentation with tumor (red), stroma (green) and necrosis/background (blue). C. WSI depicting tumor segmentation. D. Heatmap with percentage of stroma evaluated in 10x field of view resulting in the localization of the most invasive region. E. Heatmap with percentage of stroma evaluated in 10x field of view resulting in the localization of the whole tumor bed.

To measure TSR in an unbiased and fully automated way, we developed a deep learning based computational pipeline termed OTSR. OTSR scores were computed both for WT and MI in three steps: 1) segmentation of tissue into relevant tissue types, 2) estimation of the region of measurement (for WT and MI, separately), and finally, 3) measuring the stromal density based on tissue abundance within the region of measurement (Fig. 1).

In the first step, a deep learning model was employed to classify WSI patches into tumor, stroma, and background (necrosis, adipose tissue, psammoma bodies, and vessels). The custom convolutional neural network was specifically designed to provide tissue segmentation at high resolutions while taking the surrounding tissue context into account (Supplementary Figure S2). WSI tissue segmentation was subsequently achieved by classifying individual WSI image patches extracted at 20x magnification. Image patches were extracted with overlap to achieve high-resolution tissue segmentation.

In the second step, the exact region of measurement was estimated for WT and MI. To measure TSR in the MI region, we first defined the MI region computationally as the region within a 1.6 mm radius - corresponding to a 10x microscopy field of view (FoV) - with the highest tumor percentage, where the tumor percentage is measured excluding the background. To exclude regions beyond the tissue border, FOV was only considered if it was composed of more than 2/3 of non-background tissue. To measure TSR for WT, the region of measurement was based on the tissue of which the local tumor density was higher than a predetermined threshold. For smaller tumors, the threshold was dynamically altered based on the tumor size, leading to a smaller stroma margin around the tumor tissue. The threshold was chosen empirically to correspond to reference tumor bed annotations indicated by the pathologists while conducting the manual TSR measurements through Slidescore.

In the final third step, TSR was measured as the stromal density within the region of measurement, excluding the background. Similar to the manual scoring, patients were stratified in stroma-rich (stroma ≥ 50%) or stroma-poor (stroma < 50%). Additionally, to explore the optimal cutoff for TSR we determined the TSR cutoff with the highest discriminative power for OS in patients who were treated with PDS, for MI and WT. Supplementary text 1 presents a more detailed description of the OTSR pipeline.

### Immunohistochemical (IHC) Staining

CD8+, CD20+, CD68+, and CD103 + cell expression were assessed by IHC. IHC was performed on the BenchMark Ultra autostainer (Ventana Medical Systems Inc., Oro Valley, AZ, USA). Three μm thick TMA sections were generated and heated at 75 °C for 28 min followed by deparaffinization and rehydration. Deparaffinization was completed in the instrument using an EZ prep solution (Ventana Medical Systems Inc., Oro Valley, AZ, USA). Heat-induced antigen retrieval was initiated using Cell Conditioning 1 (Ventana Medical Systems Inc., Oro Valley, AZ, USA) for 32 min at 95 °C. CD8 + was detected using clone C8/144B (1/200 dilution, M7103, Agilent Technologies, Santa Clara, CA, USA), CD20 + was detected using clone L26 (1/800 dilution, M0755, Agilent Technologies, Santa Clara, CA, USA), CD68 + was detected using clone KP1 (1/20000 dilution, M0814, Agilent Technologies, Santa Clara, CA, USA). CD103 + staining was performed using anti-αEβ7-integrin (1/200 dilution, ab129202, Abcam, Cambridge, UK), as described by Komdeur et al. [26]. The bound antibodies were detected using the OptiView DAB Detection (Ventana Medical Systems Inc., Oro Valley, AZ, USA). Slides were counterstained with Hematoxylin II and Bluing reagent (Ventana Medical Systems Inc., Oro Valley, AZ, USA). All stained TMA slides were digitalized with a 20× magnification, using Leica Aperio AT2 Digital Pathology Slide Scanner (Leica Microsystems, Wetzlar, Germany). Total numbers of CD8+, CD20+, CD68+, and CD103 + cells were manually counted per core and scored as 0, 1–5, 6–19, 20–49, 50–100, or > 100 positive cells. The highest count per tumor was used. Immune cells were further categorized into low, medium, and high densities based on the 25th and 75th percentiles and the median. For CD103 + cells, only intraepithelial-located cells were counted and analyzed. All slides were counted manually by 2 individuals, differences in counts of over 10% were reanalyzed and discussed until a consensus was reached.

### Code availability

The underlying code for this study is available in github and zenodo and can be accessed via this link [https://github.com/cwlkr/OTSR] and this link [https://doi.org/10.5281/zenodo.8109931] with a persistent record.

### Statistical analysis.

Statistical analyses were performed in STATA/SE (version 14.1, STATA CORP, College Station, TX, USA). A p-value < 0.05 was considered statistically significant. Clinical associations with TSR ratios and patient characteristics were assessed with Chi-square, or Fisher’s exact test if > 20% of expected cell counts are less than 5, for categorical variables, one-way ANOVA for normally distributed continuous variables, and Kruskal–Wallis for non-normally distributed continuous variables. Kaplan–Meier survival estimates with the corresponding log-rank test and univariable and multivariable Cox regression analyses were used to assess the effect of TSR on progression-free survival (PFS) and overall survival (OS). Those found significant in univariable analyses with a p < 0.10, were included in the multivariable regression analyses and assessed using backward selection. PFS was calculated as the time between the date of diagnosis of the primary tumor and the date of recurrence. OS was calculated as the interval between the date of diagnosis of the primary tumor and the date of death. Patients without recurrence and/or alive at the date of the last check of the municipal population register (31 January 2022) were right censored for the respective analysis.

## Results

### Patient characteristics.

Tissue samples of 360 patients with FIGO stage III-IV ovarian cancer were identified. and collected (Supplementary Figure S1A), of which 344 samples were available for WSI analysis. Four samples contained too little tumor and were excluded from analysis resulting in 340 samples (Supplementary Figure S1B). 15 MI cases and 17 WT cases, respectively, were excluded from computational scoring due to too little tissue or poor image quality. After excluding the test cases, 303 computationally scored patients for MI and 301 for WT were available for analysis (Supplementary Figure S1B). Most FFPE blocks were derived from the ovary/tuba (79%). All clinicopathologic characteristics of patients are summarized in Table 1. 186 (55%) patients were 65 years or older, and 314 (92%) patients presented with FIGO stage III or higher, at diagnosis, with pleural malignant effusion being the most common distant metastasis. 141 (42%) patients underwent PDS and 199 (58%) received NACT-IDS. Complete cytoreduction was reached in 191 (56%) patients. OS and PFS data were available for all cases. Median OS and PFS were 38.3 months (Interquartile range (IQR) 22.0–69.1 months) and 17 months (11.9–37.3), respectively. 253 (74.4%) patients experienced a recurrence and 292 (86%) patients died.

### Quantifying tumor stroma composition with computational and manual image analysis

All slides were scored by two independent pathologists depicting a significant positive correlation between both scores (MI; Pearson R = 0.93, and Interclass Correlation Coefficient (ICC) = 0.93) (Supplementary Figure S3). 112 (33%) patients had a stroma-rich MI region compared to 175 (52%) WT (Table 1). OTSR resulted in 79 (26%) patients with a stroma-rich MI region compared to 150 (50%) WT. A significant positive correlation was seen between manual and OTSR scores (MI; Pearson R = 0.83 and Interclass Correlation Coefficient (ICC) = 0.81) suggesting a high correlation between both methods (Supplementary Figure S4). In patients treated with PDS the optimal computational cutoff, stroma-rich versus stroma-poor, was 18% stroma in MI and 28% stroma in WT resulting in 63% and 88% of patients with a stroma-rich MI and WT region, respectively (data not shown).

### Association between TSR and clinicopathologic characteristics.

We examined the association between TSR and all clinicopathologic characteristics. We divided TSR by therapy sequence due to the proposed effect of chemotherapy on the tumor microenvironment (Table 2).(21) In the case of PDS, stroma-rich was more frequently seen in samples taken from the omentum (36% vs 8%; rich vs poor-stroma, P < 0.001) and was more often associated with papillary tumor growth (60% vs 34%, rich vs poor-stroma, P = 0.021). A complete debulking was less often seen in stroma-rich cases with borderline significance (52% vs 78%, rich vs poor-stroma, P = 0.059). In NACT, stroma-rich was associated with a higher frequency of malignant pleural effusion (25% vs 16%; rich vs poor-stroma, P = 0.045) and lower TRG score (4&5: 21% vs 57%; rich vs poor-stroma, P < 0.001). When looking at TILs, densities differed significantly amongst TSR (Table 2). Higher densities of CD103 + and CD68 + cells (26% vs 8%, P = 0.053; 40% vs 8%, P = 0.002, respectively) were seen in a poor compared to rich-stroma in patients treated with PDS. Higher densities of CD103+ (24% vs 9%, P = 0.006), but lower CD20 + cells (12% vs 24%, P = 0.020) were seen in a poor compared to rich-stroma in patients treated NACT.

### Association between TSR and Survival.

**Figure 2:**
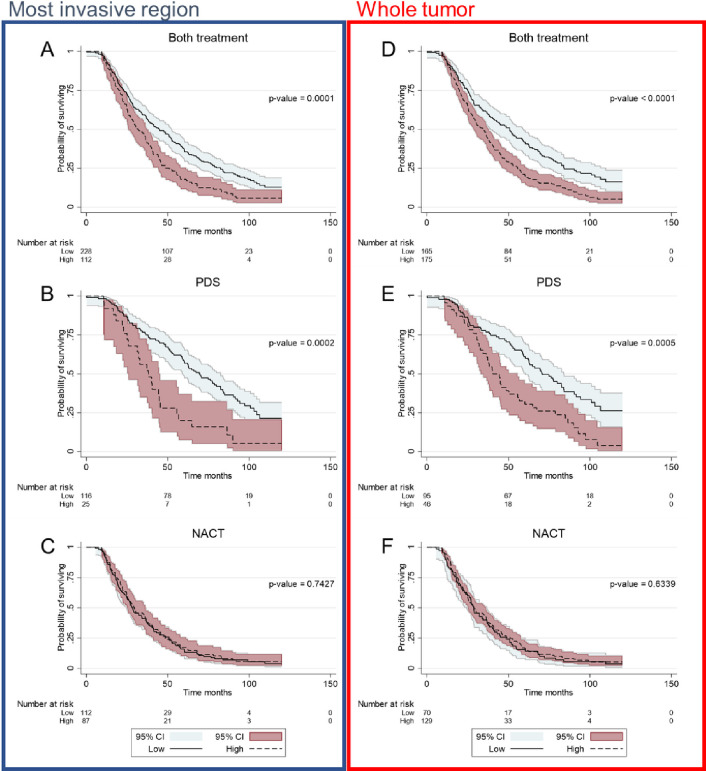
Kaplan-Meier curve of the TSR (tumor stroma ratio) in the most invasive region (MI) (blue encircled, A-C) and whole tumor region (WT) (red encircled, D-F) scored by pathologists. TSR was divided in low <50 and high ≥50% stroma. A.Patients treated with PDS & NACT in MI. B. Patients treated with PDS in MI. C. Patients treated with NACT in MI. D. Patients treated with PDS & NACT in WT. E. Patients treated with PDS in WT. F. Patients treated with NACT in WT. P values were derived with the use of the log-rank statistic. Abbreviations: 95% CI: 95% confidence interval; P: P-value; PDS: Primary debulking surgery; NACT: Neoadjuvant chemotherapy.

**Figure 3:**
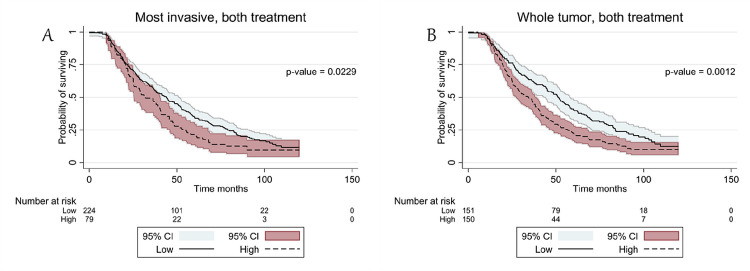
Kaplan-Meier curve of the TSR (tumor stroma ratio) in the most invasive tumor region and in the whole tumor calculated by computational pathology. TSR was divided in low <50 and high ≥50% stroma. A. Kaplan-Meier curve of the TSR in the most invasive tumor region containing patients treated with PDS & NACT. B. Kaplan-Meier curve of the TSR in the whole tumor containing patients treated with PDS & NACT. Abbreviations: 95% CI: 95% confidence interval; P: P-value; PDS: Primary debulking surgery; NACT: Neoadjuvant chemotherapy.

We observed that patients with stroma-rich tumors have significantly worse OS in both MI (Hazard ratio (HR) = 1.59, 95% Confidence Interval (CI) = 1.25–2.02, log-rank test: P = 0.001) and WT (HR = 1.64, 95% CI = 1.30–2.07, log-rank test: P < 0.001) (Table 3, Fig. 2A, Fig. 2D). This association was driven by the influence of stroma-rich tumors on survival in patients treated with PDS (MI: HR = 2.37, 95% CI = 1.48–3.80, log-rank test P = 0.0002; WT: HR = 2.01, 95% CI = 1.35–2.99, log-rank test: P = 0.0005, Supplementary Table S1, Fig. 2B Fig. 2E). In contrast, TSR was not associated with OS in patients treated with NACT (Supplementary Table S1, Fig. 2C, Fig. 2F). In the case of computationally scored TSR, stroma-rich tumors were significantly correlated with worse OS in both MI (HR = 1.37, 95% CI = 1.04–1.81, log-rank test: P = 0.0229) and WT (HR = 1.50, 95% CI = 1.17–1.92, log-rank test: P = 0.0012) (Table 3, Fig. 3A, Fig. 3B). This association was also more pronounced in cases of patients treated with PDS, but only significant in the optimal cutoff computational score (Supplementary Table S1). Similarly, stroma-rich tumors were significantly associated with a worse PFS in both MI (HR = 1.54, 95% CI = 1.20–1.95) and WT (HR = 1.57, 95% CI = 1.24–1.98) in manually scored TSR as well as in MI (HR = 1.45, 95% CI = 1.10–1.90) and WT (HR = 1.55, 95% CI = 1.21–1.98) in OTSR (Table 3).

### Independent prognostic value of TSR

To evaluate whether TSR is an independent prognostic biomarker, we performed multivariable analysis correcting for parameters that were found significant in univariable analysis (Supplementary Table S2). Known prognostic factors for ovarian cancer as the completeness of debulking, age, FIGO stage, therapy sequence, and residual status were significantly associated with OS. Notably, the localization from where the H&E slide originated, such as the ovary or the omentum, did not influence OS. When combining both treatment types, TSR remained prognostic in a multivariable analysis for OS, but only statistically significant in the case of OTSR when employing the optimal cutoff (MI; HR = 1.39, 95%CI = 1.04–1.84)(Table 3). When subgrouping for treatment type, TSR remained significantly associated with OS in multivariable analysis in the case of MI (HR = 1.83, 95%CI = 1.07–3.13) and WT (HR = 1.83, 95%CI = 1.18–2.82) (Supplementary Table S1) in manually scored cases of patients treated with PDS. OTSR did not show statistical significance in patients treated with PDS, most likely as a result of low patient numbers. In NACT, TSR was not significantly associated with OS. PFS also did not remain significantly associated with TSR in multivariable analysis (Table 3).

### Lymphocyte and stromal cell ratio as a joint prognostic classifier.

**Figure 4:**
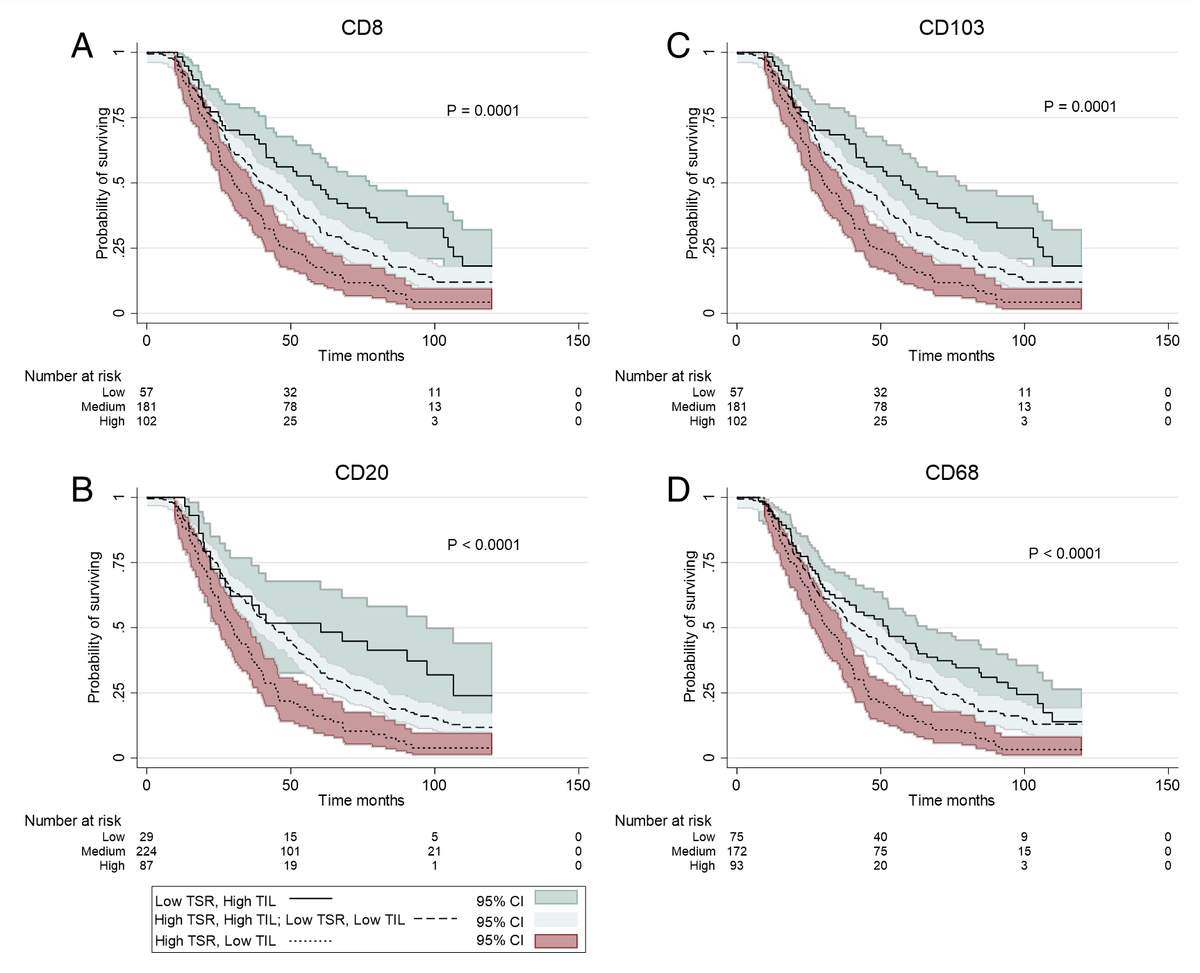
Kaplan-Meier curve of the Tumor stroma ratio (TSR) combined with Tumor infiltrating lymfocytes (TILS) generating three survival groups, 1. solid line: Low TSR and high TIL; 2. dashed line: High TSR and High TIL or Low TSR and Low TIL; and 3. dotted line: High TSR Low TIL. A. Kaplan-Meier curve of survival groups with CD8. B. Kaplan-Meier curve of survival groups with CD20. C. Kaplan-Meier curve of survival groups with CD103. D. Kaplan-Meier curve of survival groups with CD68. Abbreviations: MI: 95% CI: 95% confidence interval; P: P-value .

Given the known prognostic value of TILs (19) and the previously demonstrated prognostic value of TSR, we investigated if both parameters could define a better prognostic classifier when employed in symphony. As suggested by chi-square analysis (Table 2), there was a negative correlation between TSR and TILs (CD68: Spearman correlation coefficient - 0.17, p = 0.0013; CD103: Spearman correlation coefficient - 0.19, p = 0.0003)(data not shown). We selected patients with the highest quartile of TIL densities as the TIL high group. As shown in a previous study, patients with high TIL densities show a significantly better OS compared with the rest of the patients, with a 10-year OS probability ranging from 15%–19% (CD8 and CD103, respectively) in high TILs versus 8–9% (CD8 and CD103, respectively) in low TILs.(19) In case of TSR, the 10-year OS probability ranged from 5%–4% in case of stroma-rich to 13%–17% in case of stroma-poor (MI and WT, respectively, Fig. 2A, Fig. 2D.) To assess the possible significance of a joint classifier, patients were assigned into three groups; low risk (stroma-poor, high TIL), medium risk (stroma-rich, high TIL or stroma-poor, low TIL) or high risk (stroma-rich, Low TIL). We observed that patients with high CD8 + densities and stroma-poor (low risk) had a 10-year OS probability of 16%, compared to 11% (medium risk) and 3% (high risk) (HR = 1.35, CI = 0.97–1.87; and HR = 2.15, CI = 1.49–3.11, respectively, Supplementary Table S3, Fig. 4A). The same results were seen in the case of CD103 (10-year OS; 19% versus 11% and 4%), CD20 (10-year OS; 24% versus 12% and 3%), and CD68 (10-year OS; 14% versus 12% and 3%), all in favor of stroma-poor and high TILs (Fig. 4B–D, Supplementary Table S3). These results remained significant in multivariable analysis for CD8 and CD103 combined with TSR (Supplementary Table S3).

## Discussion

In this study, we presented the prognostic value of TSR in advanced-stage HGSOC both in the most invasive tumor region as well as in the whole tumor. We also identified a difference in the prognostic value of TSR in patients treated with PDS followed by chemotherapy versus patients treated with NACT-IDS. We additionally presented an algorithm, OSTR, which computationally quantifies TSR in both regions using H&E-stained slides. Furthermore, we demonstrated a joint prognostic classifier combining TSR and distinct immune cell densities.

There is increasing evidence supporting the importance of the TME in ovarian cancer progression.(3–5) However, most studies are performed using costly methods such as molecular profiling. We demonstrate a cost-effective way to study the TME and identify high-risk patients with routinely generated H&E slides. The present study focused on both the most invasive region and the whole tumor, thus differing from studies focusing on only one of both areas.

The key finding of our study is that the stroma-rich feature is significantly associated with a poor prognosis in HGSOC, in both the MI region as well as the WT, scored either manually or computationally. TSR can effectively separate patients into two groups, with either a favorable or unfavorable prognosis, independent of and complementary to known prognostic variables. This observation is consistent with the study by Chen *et al*., in which patients with stroma-rich in the MI region depicted a worse OS and PFS in epithelial ovarian cancer.(15) Furthermore, the results from our automated image analyses are consistent with Lan *et al*. and Jiang *et al*., who described an automated image analysis of TSR, using the WT, in which higher percentages of stromal cells were associated with worse survival.(16, 17) In addition to the previous studies, we indicated that TSR is a prognostic parameter in both the MI and WT region and that both regions can be computationally scored generating similar results to images scored by pathologists. We also depicted that the TSR is not a prognostic parameter in patients treated with NACT, which could be a result of stromal recomposition.

In daily practice, histological characteristics, such as TSR, are manually determined by individual pathologists. Manual examination of H&E stained slides is influenced by inter- and intra-observer variability (22), and relies on the pathologistś experience and expertise. An unbiased quantification is often unrealistic which could result in heterogenous results and influence reproducibility. The use of computational scoring methods, such as OTSR, eliminates inter- and intra-observer variability. OTSR also eliminates the time that the pathologist needs to dedicate to scoring the H&E slides and is therefore cost-effective. Lastly, OTSR enables the use of an optimal threshold, resulting in a more nuanced and reproducible biomarker allowing for more specific patient stratification.

Stromal density has been associated with TIL density, with higher stromal percentages being associated with lower lymphocytic infiltration.(16, 23) T-cell infiltration is a well-described prognostic marker in ovarian cancer, in which higher amounts of TILs are associated with favorable survival.(4, 24) Our study confirmed the association between TIL and stromal densities. In contrast to previous reports, we depicted distinct TILs, CD103 + and CD68+, that were significantly lower in the case of stroma-rich tumors. Interestingly, in patients treated with NACT, CD20 + densities were positively correlated with TSR, which could be a reflection of the known increase in TILs post-NACT,(25) or a TIL-specific relationship with TSR. We also demonstrated that immune cell infiltration and TSR could co-define a prognostic classifier, in agreement with previous reports.(16) Again, unlike previous studies,(16) we depicted distinct TILs, in this case, CD8 + and CD103+, that were independent prognostic markers when combined with TSR, in which stroma-rich and low TILs had a significantly worse OS compared to stroma-poor and high TILs. Taken together, our data suggest that the H&E-based TSR could be intrinsic and clinically relevant in advanced-stage ovarian cancer and could identify distinct morphological subtypes in HGSOC. Nonetheless, more studies are needed to clarify the associations between specific TILs and TSR.

The strength of the present study is that we integrated detailed pathological and computational scoring of the TME with immunological and clinical data from a large cohort of patients with HGSOC. Additionally, we predicted TSR in both the MI and WT and not only on a whole slide level as previous reports suggest that tissue regions contribute to the prognostic significance of TSR. All slides were centrally reviewed by two independent dedicated pathologists in gynecologic oncology. Lastly, a long follow-up was achieved and clinical data were complete. Limitations of the study include its historic nature, heterogeneous treatment regimen, sample size, and lack of a validation set. Furthermore, our study included samples from three separate institutions. H&E imaging-based digital pathology studies may be affected by paraffin block preservation protocols, which could lead to model differences. Nonetheless, our study showed no association between TSR scores and the institution from which the sample was derived demonstrating the robustness of the computational model. Although the prognostic value of computational and pathological scored TSR was similar, pathological expertise was essential for developing the initial OTSR. Future studies are needed to identify the influence of stromal subtypes on cancer progression which could be key to new therapeutic targets.

As stated, cancer development is influenced by the interaction between the tumor and the microenvironment.(6) As a result, the TME has become an attractive target for new therapeutic targets.(26) Our study depicted that TSR, as a part of the TME, is a cost-effective and easy-to-determine prognostic marker based on H&E-stained tumor slides that are generated during routine pathological examination. Assessment of TSR could identify patients with significantly worse prognoses who could benefit from novel agents targeting the stromal fraction of the tumor.

## Figures and Tables

**Table 1.** Patient and Tumor Characteristics

Characteristic ( N=340)			

*Tumor characteristics*		*Patient characteristics*	
**TSR; MI, pathologist**		**Age**	
High	112 (32.9%)	<65	154 (45.3%)
Low	228 (67.1%)	65–75 years	128 (37.6%)
**TSR; WT, pathologist**		>75 years	58 (17.1%)
High	175 (51.5%)	**FIGO stage**	
Low	165 (48.5%)	FIGO II	19 (5.6%)
**TSR; MI, computational** *		FIGO III	214 (62.9%)
High	79 (26.1%)	FIGO IV	100 (29.4%)
Low	224 ( 73.9%)	Unknown	7 (2.1%)
**TSR; WT, computational** **		**Therapy**	
High	150 (49.8%)	Primary debulking	141 (41.5%)
Low	151 (50.2%)	NACT	199 (58.5%)
**TMA localization**		**Residual status**	
Ovary, tuba	268 (78.8%)	Suboptimal	29 (8.5%)
Omentum, peritoneum	55 (16.2%)	Optimal	111 (32.6%)
Other	17 (5.0%)	Complete	191 (56.2%)
**CD8**		Unknown	9 (2.6%)
0–99	249 (73.2%)	**Metastases localization**	
≥100	91 (26.8%)	Pleural	44 (12.9%)
**CD103**		Lymphnodes	17 (5.0%)
0–99	273 (80.3%)	Visceral	19 (5.6%)
≥100	67 (19.7%)	Other	16 (4.7%)
**CD20**		**Ascites**	
0–49	286 (84.1%)	<100mL	209 (61.5%)
≥50	54 (15.9%)	≥100mL	83 (24.4%)
**CD68**		Unknown	48 (14.1%)
0–99	246 (72.4%)	**CA-125**	
≥100	94 (27.6%)	0–35	7 (2.1%)
		>35	257 (75.6%)
		Unknown	76 (22.4%)
		**Charlson comorbidity**	
		Charlson 0	225 (66.2%)
		Charlson 1–2	103 (30.3%)
Charlson ≥3	2 (0.6%)		
		Unknown	10 (2.9%)

* N = 301,

** N = 303

Abbreviations: TSR: tumor stroma ratio; MI: most invasive region; WT: Whole Tumor; NACT: neoadjuvant chemotherapy; IQR: Inter quartile range.

**Table 2.** Tumor and patient characteristics by most invasive TSR and treatment type

	Primairy Debulking			NACT		
Characteristic	Stroma-poor*	Stroma-rich*	P	Stroma-poor*	Stroma-rich*	P
	N = 116	N = 25		N = 112	N = 87	
**Age**						
<65	52 (44.8%)	10 (40.0%)	0.057	46 (41.1%)	46 (52.9%)	0.20
65–75 years	49 (42.2%)	7 (28.0%)		46 (41.1%)	26 (29.9%)	
>75 years	15 (12.9%)	8 (32.0%)		20 (17.9%)	15 (17.2%)	
**Figo stage**						
FIGO II	18 (15.5%)	1 (4.0%)	0.13	0 (0%)	0 (0%)	0.78
FIGO III	86 (74.1%)	19 (76.0%)		61 (54.5%)	48 (55.2%)	
FIGO IV	8 (6.9%)	4 (16.0%)		51 (45.5%)	37 (42.5%)	
Unknown	4 (3.4%)	1 (4.0%)		0 (0.0%)	2 (2.3%)	
**Residual disease**						
Suboptimal	7 (6.0%)	2 (8.0%)	**0.059**	11 (9.8%)	9 (10.3%)	0.99
Optimal	14 (12.1%)	7 (28.0%)		51 (45.5%)	39 (44.8%)	
Complete	90 (77.6%)	13 (52.0%)		49 (43.8%)	39 (44.8%)	
Unknown	5 (4.3%)	3 (12.0%)		1 (0.9%)	0 (0.0%)	
**TMA localization**						
Ovary, tuba	104 (89.7%)	14 (56.0%)	**<0.001**	88 (78.6%)	62 (71.3%)	0.37
Omentum, peritoneum	9 (7.8%)	9 (36.0%)		17 (15.2%)	20 (23.0%)	
Other	3 (2.6%)	2 (8.0%)		7 (6.3%)	5 (5.7%)	
**Metastases localization**						
Pleural	2 (1.7%)	2 (8.0%)	**0.76**	18 (16.1%)	22 (25.3%)	**0.045**
Lymphnodes	2 (1.7%)	1 (4.0%)		8 (7.1%)	6 (6.9%)	
Visceral	3 (2.6%)	1 (4.0%)		13 (11.6%)	2 (2.3%)	
Unknown	0 (0%)	0 (0%)		9 (8.0%)	7 (8.0%)	
No metastasis	109 (94.0%)	21 (84.0%)		64 (57.1%)	50 (57.5%)	
**Growth Pattern tumor**						
Papillar	39 (33.6%)	15 (60.0%)	**0.021**	36 (32.1%)	29 (33.3%)	0.17
Solid	60 (51.7%)	5 (20.0%)		69 (61.6%)	46 (52.9%)	
Glandular	6 (5.2%)	3 (12.0%)		4 (3.6%)	10 (11.5%)	
Other	11 (9.5%)	2 (8.0%)		3 (2.7%)	2 (2.3%)	
**Mandart score**	N.A.					
1 & 2				11 (9.8%)	30 (34.5%)	**<0.001**
3				33 (29.5%)	36 (41.4%)	
4 & 5				64 (57.1%)	18 (20.7%)	
Unknown				4 (3.6%)	3 (3.4%)		
**CD8**						
0–99	88 (75.9%)	19 (76.0%)	0.99	79 (70.5%)	63 (72.4%)	0.77
≥100	28 (24.1%)	6 (24.0%)		33 (29.5%)	24 (27.6%)	
**CD103**						
0–99	86 (74.1%)	23 (92.0%)	**0.053**	85 (75.9%)	79 (90.8%)	**0.006**
≥100	30 (25.9%)	2 (8.0%)		27 (24.1%)	8 (9.2%)	
**CD20**						
0–49	100 (86.2%)	21 (84.0%)	0.77	99 (88.4%)	66 (75.9%)	**0.020**
≥50	16 (13.8%)	4 (16.0%)		13 (11.6%)	21 (24.1%)	
**CD68**						
0–99	70 (60.3%)	23 (92.0%)	**0.002**	83 (74.1%)	70 (80.5%)	0.29
≥100	46 (39.7%)	2 (8.0%)		29 (25.9%)	17 (19.5%)	

* Based on most invasive TSR scored by pathologists. Abbreviations TSR: tumor stroma ratio; NACT: neoadjuvant chemotherapy; TMA: Tumor micro array; P: P-value

**Table 3.** Survival analysis, Tumor stroma ratio

	OS (N = 340 - Events = 292 - Median = 38.3 [22.0–69.1])					PFS (N = 340 - Events = 253 - Median = 16.8 [11.9–37.3])				
	Median survival, months (IQR)	Crude HR (95% CI)	P	Adjusted HR* (95% CI)	P	Median Progression Free survival, months (IQR)	Crude HR (95% CI)	P	Adjusted HR* (95% CI)	P
**Most invasive**										
Stroma-poor	44.6 (23–82)	REF		REF		18.4 (12.4–46.3)	REF		REF	
Stroma-rich	31.3 (20–49)	1.59 (1.25–2.02)	**<0.0001**	1.07 (0.82–1.39)	0.606	15.1 (11.2–23.7)	1.59 (1.23–2.06)	**<0.0001**	1.01 (0.76–1.33)	0.954
**Whole tumor**										
Stroma-poor	50.8 (25–84)	REF		REF		20.3 (12.5–52.6)	REF		REF	
Stroma-rich	34.4 (20–56)	1.64 (1.30–2.07)	**<0.0001**	1.22 (0.94–1.57)	0.135	15.2 (11.6–24.8)	1.75 (1.36–2.26)	**<0.0001**	1.15 (0.88–1.52)	0.310
**Most invasive, computational**										
Stroma-poor	44.0 (22–79)	REF		REF		18.4 (12.1–42.2)	REF		REF	
Stroma-rich	32.1 (21–55)	1.37 (1.04–1.81)	**0.024**	0.98 (0.73–1.33)	0.912	15.1 (11.2–23.7)	1.54 (1.15–2.05)	**0.003**	0.93 (0.68–1.26)	0.634
**Whole tumor, computational**										
Stroma-poor	51.3 (25–84)	REF		REF		24.7 (13.2–47.2)	REF		REF	
Stroma-rich	34.1 (20–57)	1.50 (1.17–1.92)	**0.001**	1.11 (0.85–1.45)	0.455	15.1 (11.2–24.0)	1.65 (1.26–2.16)	**<0.0001**	1.05 (0.78–1.40)	0.765
**Most invasive, computational, optimal cutoff**										
Stroma-poor	57.0 (27101)	REF		REF		26.8 (14.0–49.7)	REF		REF	
Stroma-rich	33.4 (19–56)	1.81 (1.39–2.34)	**<0.0001**	1.39 (1.04–1.84)	0.024	14.1 (11.4–25.3)	1.80 (1.35–2.40)	**<0.0001**	1.15 (0.84–1.56)	0.386
**Whole tumor, computational, optimal cutoff**										
Stroma-poor	70.7 (34-x)	REF		REF		37.4 (15.9–60.1)	REF		REF	
Stroma-rich	37.5 (21–65)	2.06 (1.35–3.15)	**0.001**	1.50 (0.96–2.34)	0.072	16.2 (11.7–32.2)	1.97 (1.24–3.13)	**0.004**	1.27 (0.78–2.07)	0.334

* Data was adjusted for age, FIGO stage, therapy sequence and outcome of surgery

Abbreviations: OS: overall survival; PFS: progression free survival; HR: Hazard Ratio; 95% CI: 95% confidence interval; IQR: interquartile range; REF: reference class; P: P-value.

## Data Availability

Python code for performing the proposed methodology and reproducing reported results are available from https://github.com/cwlkr/OTSR with a persistent record on https://doi.org/10.5281/zenodo.8109931. Data sharing of anonymous clinical data from the NCR will be considered for non-commercial, research, or statistical-based use on a case-by-case basis (to be requested and approved by the NCR; gegevensaanvraag@iknl.nl). The human sequence data and tumor tissue data generated in this study are not publicly available due to patient privacy requirements but are available upon reasonable request from the corresponding author.
